# Evaluation of the effects of a standardized aqueous extract of *Phyllanthus emblica* fruits on endothelial dysfunction, oxidative stress, systemic inflammation and lipid profile in subjects with metabolic syndrome: a randomised, double blind, placebo controlled clinical study

**DOI:** 10.1186/s12906-019-2509-5

**Published:** 2019-05-06

**Authors:** Pingali Usharani, Padma Latha Merugu, Chandrasekhar Nutalapati

**Affiliations:** Department of Pharmacology and Therapeutics, Nizam’s Institute of Medical Sciences, Telangana, India

**Keywords:** *Phyllanthus emblica*, *Emblica officinalis*, Amla, Reflection index, Dyslipidemia, Oxidative stress, Systemic inflammation, Endothelial dysfunction

## Abstract

**Background:**

Endothelial dysfunction (ED) has been observed in individuals with metabolic syndrome (MetS) and contributes to the initiation and progression of atherosclerosis. The primary management of MetS involves lifestyle modifications and treatment of its individual components with drugs all of which have side effects. Thus, it would be of advantageous if natural products would be used as adjuncts or substitutes for conventional drugs. The aim of the present study was to evaluate the effect of standardized aqueous extract of fruits of *Phyllanthus emblica (P. emblica)* 250 mg and 500 mg twice daily on ED, oxidative stress, systemic inflammation and lipid profile in subjects with MetS.

**Methods:**

In this randomised, double-blind, placebo-controlled clinical study endothelial function was measured by calculating reflection index (RI) using digital plethysmograph. Oxidative stress biomarkers used were nitric oxide (NO), glutathione (GSH) and malondialdehyde (MDA). Systemic inflammation was measured by determining high sensitivity C-reactive protein (hsCRP) and dyslipidemia by lipid profile. ANOVA, paired and unpaired t-test were used. *P*-value < 0.05 was considered statistically significant.

**Results:**

Out of 65 screened subjects all 59 enrolled completed the study. *P. emblica* aqueous extract (PEE), 250 mg and 500 mg twice daily dosing, showed significant reduction in mean RI, measure of endothelial function, at 8 and 12 weeks (*p* <  0.001) compared to baseline and placebo. Significant mean % change was seen in oxidative stress biomarkers, NO (+ 41.89%, + 50.7%), GSH (+ 24.31%, + 53.22%) and MDA (− 21.02%, − 31.44%), and systemic inflammation biomarker, hsCRP (− 39.68%, − 53.77%) (*p* <  0.001) at 12 weeks with 250 mg and 500 mg twice daily dosage respectively. Significant mean % change was also seen at 12 weeks with TC (− 7.71%, − 11.11%), HDL-C (+ 7.33% + 22.16%, *p* <  0.05), LDL-C (− 11.39%, − 21.8%) and TG (− 9.81%, − 19.22%) respectively with 250 mg and 500 mg twice daily (*p* <  0.001). *PEE* 500 mg twice daily was significantly more efficacious than the 250 mg twice daily and placebo. No participant discontinued the study because of adverse events.

**Conclusions:**

*P.emblica* aqueous extract significantly improved endothelial function, oxidative stress, systemic inflammation and lipid profile at both dosages tested, but especially at 500 mg twice daily. Thus, this product may be used as an adjunct to conventional therapy (lifestyle modification and pharmacological intervention) in the management of metabolic syndrome.

**Trial registration:**

This study was registered with Clinical Trials Registry – India (CTRI) with the registration number of CTRI/2017/09/009606. The study was registered retrospectively on 4th September 2017.

## Background

Metabolic syndrome (MetS) is characterized by the coexistence of multiple interrelated cardiovascular (CV) risk factors in a single individual [1]. The National Cholesterol Education Program’s Adult Treatment Panel III (ATP III) identified six components of the MetS: abdominal obesity, insulin resistance with or without glucose intolerance, atherogenic dyslipidaemia, raised blood pressure, prothrombotic state, and proinflammatory state [2]. MetS affects 25% of the world population, and its incidence is gradually increasing [3].

Endothelial dysfunction (ED) is abnormal functioning of the endothelial cells, caused by a decrease in bioavailability of vasodilator substances, particularly nitric oxide (NO), and increase of vasoconstrictor substances. Numerous metabolic abnormalities found in MetS result in endothelial cell dysfunction by affecting NO synthesis or degradation. The frequent metabolic abnormalities that may contribute to ED in patients with MetS are hyperinsulinemia, hyperglycaemia, increase in the levels of fatty acids, C- reactive protein, oxidative stress, triglycerides and low-density lipoprotein cholesterol (LDL-C), and a decrease in high-density lipoprotein cholesterol (HDL-C) [4].

Both obesity and MetS have been independently associated with increased oxidative and inflammatory stress [5]. Oxidative stress is due to the production of reactive oxygen species (ROS), increased lipid peroxidation and impairment of antioxidant enzymatic defences, such as superoxide dismutase (SOD) or glutathione peroxidase (GPx) or combination of these factors [6]. Lipid peroxidation leads to the formation of malondialdehyde (MDA), and increased levels of MDA are found in obesity and insulin resistance. MDA can induce the increased expression of pro-inflammatory cytokines, resulting in systemic stress [7]. C-reactive protein (CRP) is a marker of systemic inflammation and a predictor of future atherosclerotic events [5]. Specifically, high sensitivity C - reactive protein (hsCRP) is an independent predictor of coronary heart disease [8].

Endothelial dysfunction (ED) is the earliest detectable functional disturbance in the natural history of the disease and a powerful predictor of future CV events [9]. Therapeutic interventions aimed at reducing CV risk factors may improve the endothelial function [10]. The primary management of MetS involves lifestyle modifications and treatment of its individual components, which includes lipid-lowering drugs, angiotensin-converting enzyme inhibitors, antidiabetic drugs, antiplatelet and antioxidant agents. However, the currently available pharmacologic interventions sometimes may not sufficiently improve endothelial function or may have side effects [11]. So, alternative treatments are needed to prevent the development of cardiovascular diseases (CVD) in subjects with MetS.

*Phllanthus emblica* (*Emblica officinalis*, Amla) fruits are an important dietary source of polyphenolic compounds, which are low molecular weight hydrolysable tannins. This fruit extract has been reported to exhibit hypolipidemic, antidiabetic, anti-inflammatory, and antioxidant properties [12]. It has been reported that the tannoid principles of aqueous extract of Amla are a potent inhibitor of lipid peroxide generation and a scavenger of hydroxyl and superoxide radicals in vitro and in vivo [13].

Clinical studies provide evidence that *P.emblica* extract has significantly improved endothelial function and reduced biomarkers of oxidative stress and systemic inflammation in patients with type 2 diabetes mellitus (T2DM) [14, 15]. As there is a paucity of data on the effect of *P.emblica* on ED in MetS, the present study was undertaken to evaluate the effect of a standardized aqueous extract of *P.emblica*, at 250 mg (PEE250) and 500 mg (PEE500) versus placebo twice daily on ED, oxidative stress, systemic inflammation and lipid profile in MetS.

## Methods

The present study was prospective, randomised, double-blind and placebo-controlled with a 12-week duration, conducted in the Department of Clinical Pharmacology and Therapeutics, NIMS, Hyderabad, India, after written approval from the Institutional Ethics Committee. Written informed consent was taken from all the subjects prior to participation in the study.

Subjects included in the study were of either gender, aged 30–68 years, having ED defined as ≤6% change in reflection index (RI) on post salbutamol challenge test and having MetS according to “The International Diabetes Federation” guidelines, dated 2006 [3]. Exclusion criteria consisted of subjects with severe uncontrolled hypertension, uncontrolled hyperglycemia, impaired hepatic or renal function, cardiac arrhythmia, chronic obstructive pulmonary disease (COPD), bronchial asthma, history of smoking, chronic alcoholism, malignancy or stroke and any other serious disease requiring active treatment and treatment with any other herbal supplements. A total of 65 subjects were screened and 59 eligible subjects were randomised by computer generated block randomisation.

**Fig. 1:**
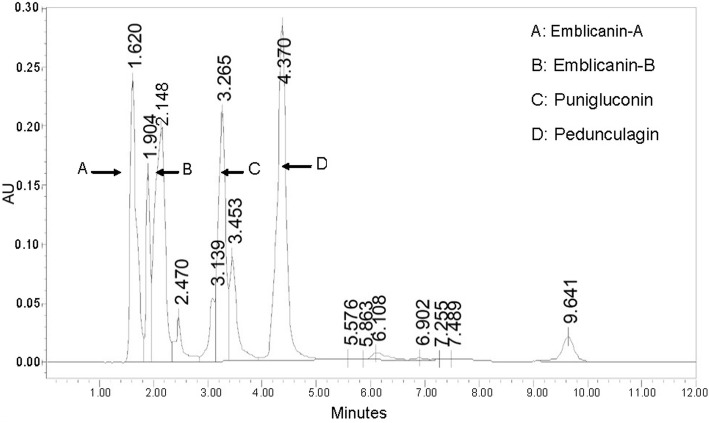
HPLC chromatogram of Capros®

Study medications: The test products, PEE250 (CAPROS® 250) and PEE500 (CAPROS® 500) and matching placebo capsules used in the present study were supplied by Natreon Inc., USA. Each capsule of the PEE250 and PEE500 comprised of aqueous extract of the edible fruits of *P. emblica*, standardized by high performance liquid chromatography (HPLC) to contain not less than 60% of low molecular weight hydrolysable tannins ((LMwHTs), comprising of Emblicanin–A, Emblicanin-B, Pedunculagin and Punigluconin as bioactives. Placebo capsules contained microcrystalline cellulose, lactose and magnesium stearate as excipients. The HPLC chromatogram of CAPROS® is shown in Fig. 1. The HPLC method used was the company’s In-house Monograph method. Briefly, 50 mg of *P.emblica* extract was dissolved in 50 ml of distilled water, and filtered through 0.22 μm syringe filter. The filtered solution (20 μL) was injected into Waters HPLC system (equipped with e2695 separation module, Photodiode Array detector (2998), and Empower3 pro Software). Compounds were separated on a NovaPak RP C_18_ 150 × 3.9 mm, 4 μ (Waters corporation, WAT086344), column using 0.1 M sodium acetate–acetic acid buffer (pH 3.9) as the mobile phase at the flow rate of 0.6 ml/min and detection wavelength 280 nm. The percentage content of the LMwHTs was calculated using area of the LMwHTs peaks and the linear regression equation of the external standard.

Eligible subjects were enrolled and randomised by the investigator. The containers containing drugs were sequentially numbered and were dispensed by the pharmacist to the subjects according to the randomly allocated sequence so as to receive one capsule of PEE250, PEE500 or placebo twice daily for 12 weeks. Subjects returned for follow up visits at 4, 8 and 12 weeks of therapy, when subjects were evaluated for efficacy and safety. Pharmacodynamic evaluation of endothelial function (RI) was conducted at every visit. A 10 ml blood sample was collected in plain blood collection tubes after an overnight fast of 12 h for evaluation of NO, MDA, glutathione (GSH), hsCRP and lipid profile at baseline and at the end of 12 weeks of treatment. A complete physical examination and safety lab investigations for hematological, hepatic and renal biochemical parameters were conducted at baseline and at the end of the study, and as required during the study. Subjects were enquired for the presence of adverse drug reaction (ADR) at every visit, and any reported ADRs were recorded in the case report form. Compliance with therapy was assessed by pill count method.

Endothelial function was evaluated by salbutamol challenge test using the digital volume plethysmography (DPG) as reported by Chowienczyk et al. and Naidu et al. [16, 17]. Subjects were examined in supine position after 5 min of rest. A digital volume pulse (DVP) was obtained using photo plethysmograph (Pulse Trace PCA2, PT200, Micro Medical, Kent, UK) transmitting infra-red light at 940 nm, placed on the index finger of right hand. The signal from the plethysmograph was digitized using a 12 bit analogue to digital converter with a sampling frequency of 100 Hz. DVP waveforms were recorded over 20 s period and the height of the late systolic / early diastolic portion of the DVP was expressed as a percentage of the amplitude of the DVP to yield the RI, as per the procedure described in detail by Millasseau et al. [18]. After DVP recordings had been taken, three measurements of RI were calculated and the mean value was determined. Subjects were then administered 400 μg of salbutamol by inhalation. After 15 min, three measurements of RI were obtained again and the difference in mean RI before and after administration of salbutamol was used for assessing endothelial function. A change of ≤6% in RI post salbutamol was considered as endothelial dysfunction.

A spectrophotometer was used to estimate serum MDA (Thiobarbituric acid reactive substance test), NO (Colorimetric detection with Griess reagents) and GSH (Ellman’s method) levels [19–21].

Peripheral venous blood was collected in plain blood collection tubes after an overnight fast of 12 h after the last dose of medication for the estimation of haemoglobin, blood urea, serum creatinine, liver function test, lipid profile using standard techniques.

### Primary and secondary efficacy parameters

The primary efficacy criterion was more than 6% change in RI (a measure of ED), assessed at 4,8 and 12 weeks. Secondary efficacy parameters included improvements in oxidative stress markers - NO, GSH and MDA, systemic inflammation marker hsCRP and the lipid profile at 12 weeks. Safety and tolerability assessment of the test medications were also conducted at 12 weeks.

### Statistical analysis

#### Sample size calculation

Sample size calculation was done based on a previous study by Nishat Fatima et.al [14]. A sample size of 65 was estimated to recruit 59 patients considering a difference of 0.91% change in mean reflection index between PEE250 versus PEE500, with anticipated standard deviation of 0.92 at 5% level of significance and 80% power of study with screen failure rate of 10% and dropout rate of 15%.

Data were expressed as mean ± SD. The Kolmogorov-Smirnov test was used to assess the normality of the data. Unpaired t-test was performed to determine the statistical significance between groups and paired t-test was used to analyse within the group. One-way ANOVA was done for between groups analysis for absolute and mean percentage change. Posthoc analysis was done by Tukey’s test. A *p*-value of less than 0.05 was considered to be statistically significant. All statistical analysis was performed using the GraphPad Prism version 4.

## Results

Out of the 65 screened subjects, 59 eligible subjects were randomised and all completed the study. Of the 59 subjects, 18 subjects received placebo, 20 subjects received PEE250 and 21 subjects received PEE500 twice daily. The subjects’ baseline characteristics are shown in Table 1, which indicate the study population was homogenous. Lipid abnormalities were present in all subjects.

**Table 1:** Baseline characteristics of subjects receiving Placebo, *Phyllanthus emblica* 250 mg and *Phyllanthus emblica* 500 mg

Parameter		Placebo (A) (*n* = 18)	*Phyllanthus emblica* 250 mg (B) (*n* = 20)	*Phyllanthus emblica* 500 mg (C) (*n* = 21)
Age in Yrs.		56.89 ± 7.39	57.45 ± 7.44	57.24 ± 8.94
Gender (M/F)		14/4	14/6	15/6
Bodyweight (Kg)		78.17 ± 5.94	78.9 ± 6.61	78.9 ± 4.87
Height (cm)		161.17 ± 2.77	162.20 ± 3.53	162.14 ± 2.83
BMI (Kg/m^2^)		30.11 ± 1.72	30.02 ± 1.66	30.05 ± 1.70
BMI (kg/m^2^_)_	< 30	5 (28%)	7 (35%)	6 (29%)
	> 30	13 (72%)	13 (65%)	15 (71%)
SBP (mmHg)	< 130	5 (28%)	7 (35%)	9 (43%)
	> 130	13 (72%)	13 (65%)	12(57%)
DBP (mmHg)	< 85	10(56%)	12(60%)	8(38%)
	> 85	8 (44%)	8 (40%)	13 (62%)
FPG (mg /dL)	< 100	13 (72%)	11 (55%)	8(38%)
	> 100	5 (28%)	9 (45%)	13 (62%)

Values expressed as mean ± SD

*SD* standard deviation, *BMI* Body Mass Index, *SBP* Systolic blood pressure, *DBP* Diastolic blood pressure, *FPG* Fasting plasma glucose

The RI was used to assess endothelial function. It can be seen from Table 2, that baseline RI was nonsignificant between the three treatments. Treatment with PEE250 and PEE500 twice daily showed a significant reduction in mean RI at 8 and 12 weeks, indicating an improvement in endothelial function (*p* <  0.001). On further analysis, it was found that change in mean RI with PEE500 was significantly better compared to PEE250 (*p* <  0.05). Compared to placebo absolute change of RI was statistically significant with the two active treatments after 8 and 12 weeks of treatment.

**Table 2:** Effect of Placebo,* Phyllanthus emblica *250 mg and * Phyllanthus emblica* 500 mg on reflection index % (marker of endothelial function)

Parameter	Placebo (A) (*n* = 18)				* Phyllanthus emblica* 250 mg (B) (*n* = 20)				* Phyllanthus emblica* 500 mg (C) (n = 21)			
RI (%)	Baseline	After treatment			Baseline	After treatment			Baseline	After treatment		
		4 weeks	8 weeks	12 weeks		4 weeks	8 weeks	12 weeks		4 weeks	8 weeks	12 weeks
Mean ± SD	−2.27 ± 1.22	−1.88 ± 1.24	− 1.46 ± 1.61	− 0.97 ± 2.45	− 2.20 ± 0.97	− 2.39 ± 1.16	− 4.18 ± 0.87*†	−8.82 ± 1.54*†	−2.52 ± 1.13	−2.76 ± 0.99	− 4.86 ± 0.88*†‡	− 10.03 ± 1.58*†‡
p		NS	NS	NS		NS	< 0.001	< 0.001		NS	< 0.001	< 0.001

Values expressed as mean ± SD

*SD* standard deviation, *RI* Reflection index

*p < 0.001: compared to baseline value. ^†^*p* < 0.001: compared to placebo (A vs B, A vs C), ^‡^ *p* < 0.05: B vs C

The mean percent change in biomarker values was analysed for each of the study treatments at 12 weeks. Significant mean % change was seen in oxidative stress biomarkers, NO (+ 41.89%, + 50.7%), GSH (+ 24.31%, + 53.22%) and MDA (− 21.02%, − 31.44%), and the systemic inflammation biomarker, hsCRP (− 39.68%, − 53.77%) (*p* <  0.001) at 12 weeks with the 250 mg and 500 mg twice daily dosage respectively (Table 3), evidence of improved endothelial function and decreased systemic inflammation. PEE500 was significantly (*p* <  0.01 to *p* < 0.001) more efficacious than PEE250.

**Table 3:** Effect of Placebo, *Phyllanthus emblica* 250 mg and *Phyllanthus emblica* 500 mg on biomarkers

Parameter	Placebo (A) (n = 18)			*Phyllanthus emblica* 250 mg (B) (n = 20)			*Phyllanthus emblica* 500 mg (C) (*n* = 21)		
	Baseline	12 weeks	Mean % change	Baseline	12 weeks	Mean % change	Baseline	12 weeks	Mean% change
NO (μM/L)	32.97 ± 4.02	32.58 ± 3.88	−0.94%	34.53 ± 5.81	47.88 ± 5.62 ^*†^	41.89%^†^	32.62 ± 3.91	48.80 ± 5.97 ^*†^	50.70%^†∞^
GSH (μM/L)	421.24 ± 61.1	429.3 ± 62.4	2.15%	424.91 ± 103.16	524.05 ± 121.5^*§^	24.31%^†^	395.07 ± 58.03	599.33 ± 62.69^*†^	53.22%^†∞^
MDA (nM/ml)	3.79 ± 0.67	3.90 ± 0.60	3.53%	3.86 ± 0.75	2.94 ± 0.57^*†^	−21.02%^†^	3.85 ± 0.79	2.48 ± 0.38^*†^	−31.44% ^†Ns^
hsCRP (mg/L)	3.61 ± 0.74	3.66 ± 0.61	2.48%	3.39 ± 0.74	2.06 ± 0.93^*†^	−39.68%^†^	3.51 ± 0.91	1.56 ± 0.63^*†^	−53.77%^†‡^

Values expressed as mean ± SD

*SD* Standard deviation, *NO* Nitric oxide, *GSH* Glutathione, *MDA* Malondialdehyde, *hsCRP* high sensitivity C - reactive protein

^*^p < 0.001 compared to baseline value. ^†^*p* < 0.001 and ^§^*p* < 0.01 compared to placebo (A vs B, A vs C). ^∞^*p* < 0.001 B vs C, ^‡^ *p* < 0.05: B vs C

Significant mean % changes were also seen at 12 weeks with TC (− 7.71%, − 11.11%), HDL-C (+ 7.33%, *p* < 0.05, + 22.16%), LDL-C (− 11.39%, − 21.8%) and TG (− 9.81%, − 19.22%) respectively with PEE250 and PEE500 twice daily dosing (p < 0.001) (Table 4). Compared to PEE250 twice daily, treatment with PEE500 twice daily was more efficacious in improving TC (*p* < 0.05), LDL-C (*p* < 0.001), HDL-C (*p* < 0.001) and TG (*p* < 0.01) levels (Table 4).

**Table 4:** Effect of Placebo*, Phyllanthus emblica* 250 mg and *Phyllanthus emblica* 500 mg on lipid profile

Parameter	Placebo (A) (*n* = 18)			*Phyllanthus emblica* 250 mg (B) (*n* = 20)			*Phyllanthus emblica* 500 mg (C) (*n* = 21)		
	Baseline	12 weeks	Mean % change	Baseline	12 weeks	Mean % change	Baseline	12 weeks 12 weeks	Mean % change
TC (mg/dl)	179.16 ± 18.2	184.16 ± 20.8	2.78%	178.9 ± 21	165.7 ± 17.3^*§^	−7.17% ^†^	183.48 ± 23.8	162.5 ± 18.9 ^*†^	−11.11% ^†‡^
HDL-C (mg/dl)	33.28 ± 2.74	33.29 ± 2.79	0.57%	32.75 ± 2.34	35.10 ± 4.24^||¶^	7.33% ^¶^	31.57 ± 2.44	38.48 ± 3.28 ^*†^	22.16%^†∞^
LDL-C (mg/dl)	136.7 ± 16.02	141.6 ± 14.79	3.83%	133.2 ± 18.31	118.1 ± 17.9^*†^	−11.39%^†^	134.86 ± 12.24	105.52 ± 14.17^*†^	−21.80%^†∞^
TG (mg/dl)	184.3 ± 19.82	184.7 ± 21.62	0.18%	184.4 ± 28.05	165.9 ± 23.43^*¶^	−9.81%^†^	198.19 ± 31.84	157.24 ± 18.12^*†^	−19.22% ^†£^

Values expressed as mean ± SD

*SD* Standard deviation, *TC* Total cholesterol, *HDL-C* High-density lipoprotein Cholesterol, *LDL-C* low-density lipoprotein cholesterol, *TG* Triglycerides

^*^p < 0.001, ^||^*p* < 0.05 compared to baseline value. ^†^*p* < 0.001, ^§^*p* < 0.01, ^¶^ *p* < 0.05 compared to placebo (A vs B, A vs C).^∞^*p* < 0.001 B vs C, ^£^*p* < 0.01 B vs C, ^‡^ *p* < 0.05: B vs C

There were no significant changes in mean heart rate, haematological, renal and hepatic functions with all treatments. All subjects tolerated therapy well. There were no serious adverse events recorded in the study. Two subjects each in the PEE250 and PEE500 groups complained of dyspepsia, and mild diarrhoea was noted in three subjects of the placebo group. All ADRs resolved with symptomatic treatment. None of the patients discontinued the study prematurely because of these adverse events.

## Discussion

In our study on 59 subjects the aqueous extract of *P.emblica* has shown statistically significant improvement in endothelial function, biomarkers of oxidative stress (NO, GSH, MDA), inflammatory marker (hsCRP) and lipid profile.

Studies by Hyun Young Kim et al. [22] and Manoj Gupta et al. [23] showed beneficial effects of *P.emblica* on parameters of MetS like oxidative stress biomarkers, lipid profile, and inflammatory markers. However, these studies did not evaluate endothelial function. In our study, RI was measured to assess endothelial function, using salbutamol challenge test employing DPG. Salbutamol challenge test used to assess endothelial function (RI) is a simple, non-invasive, reproducible and a reliable method [17]. This test does not require trained personnel unlike flow-mediated vasodilation (FMD) which requires sophisticated instrument, a skilled operator and has a chance for large inter-individual variability in the assessment of ED [17, 24]. In our earlier study using salbutamol challenge test, we have reported a significant reduction in mean RI with *P.emblica* 250 mg (from − 2.25 ± 1.37 to − 9.13 ± 2.56) and 500 mg (from − 2.11 ± 0.98 to − 10.04 ± 0.92) after 12 weeks of treatment in patients with T2DM [14]. In another study, we demonstrated a significant reduction in the mean RI compared to baseline and placebo with *Terminalia chebula* 250 mg [from − 2.25 ± 0.70 to − 3.72 ± 1.35 mg] and 500 mg [from − 2.35 ± 0.85 to − 6.14 ± 1.01] in MetS subjects [25]. Keisuke Fujitakaa et al. in their study reported that administration of modified resveratrol (Longevinex) for 3 months improves endothelial function (measured using flow-mediated dilatation) in adults with MetS receiving standard treatment for T2DM, dyslipidaemia, or hypertension [26]. Results of the earlier mentioned study [14] are in accordance with our study where we found that treatment with a standardized aqueous extract of the edible fruits of *P. emblica* showed a significant reduction in mean RI compared to baseline and placebo using DPG method.

Increased oxidative stress plays a pivotal role in the pathogenesis of ED. Tung-Sheng Chen et al., have reported that oral administration of a 1:1 mixture of epigallocatechin gallate (EGCG) and *Emblica officinalis* extract 300 mg/day for 3 months significantly reduced the MDA (*p* < 0.001) levels in diabetic uremic patients [27]. Hyun Young Kim et al. demonstrated that oral administration of an ethyl acetate (EtOAc) extract of amla, to high-fructose diet fed rats at the dose of 20 mg/kg reduced the serum and hepatic mitochondrial MDA levels (p < 0.001) [22]. Our study had shown results similar to Tung- Sheng Chen et al. We found that 12 week therapy with PEE250 and PEE500 significantly decreased MDA levels compared to baseline and placebo. Additionally, we also evaluated NO and GSH and reported a statistically significant increase in their levels. In an earlier study done by us with *P. emblica* on T2DM patients, we reported similar results on biomarkers of oxidative stress [14]. No significant changes were observed in the placebo group in any of these evaluated parameters.

Akhtar et al. reported that treatment with *Emblica officinalis* fruit powder, either 2 or 3 g per day for 21 days, significantly increased HDL-C (*p* < 0.05) and lowered TC (p < 0.05) and TG (p < 0.05), LDL-C (p < 0.05) levels in normal and diabetic human subjects [28]. Savita Khanna et al. demonstrated that 3 months oral supplementation of standardized extract of *P. emblica* fruit 500 mg twice daily, showed a significant decrease in LDL-C (*p* = 0.023) and total cholesterol/HDL ratio (*p* = 0.006) in overweight/Class-1 obese adults [8]. A pilot study done by Biswas et al. in human volunteers with a long smoking history, demonstrated a marginal decrease in cholesterol (− 6.1%), triglyceride (− 3.2%) and LDL (− 9.4%) but a significant increase in serum HDL level (25.6%) with *Emblica officinalis* extract [29], the same extract used in the present study. Our results were in accordance with the reported study [29] and we also found that 12 weeks of treatment with PEE250 and PEE500 twice daily showed a significant change in TC (− 7.71% and − 11.11%), LDL-C (− 11.39% and − 21.8%), and TG (− 9.81% and − 19.22%) and HDL-C (+ 7.33% and + 22.16%) compared to baseline for PEE250 and PEE500 respectively. PEE500 was significantly more efficacious than PEE250, while the placebo group had no significant effect on any of the parameters. The improvement in lipid parameters which were found in our study may be attributed to the fact that hypercholesterolemia induces a number of changes in vascular homeostasis and it appears that cholesterol-induced ED is linked to the degree of LDL oxidation and not LDL concentration itself [30]. Antioxidants may be protective against oxidative stress and preserve NO by scavenging ROS and inhibiting oxidized LDL (OxLDL) formation [31].

Systemic inflammation biomarkers, hsCRP, is a predictor of CV events in healthy individuals as well as in persons with the MetS [32]. Antony et al. observed that treatment with Amlamax®, at doses of 500 mg/day and 1000 mg/day, brought about a significant fall in blood CRP levels [33]. Savita Khanna et al. demonstrated that treatment with standardized *P.emblica* fruit extract, 500 mg twice daily for 3 months, revealed a significant decrease in hsCRP (*p* < 0.001) in overweight /Class-1 obese adults [8]. In another study, it was observed that there was a significant change (− 14.3%) in the level of hsCRP levels with *Emblica officinalis* extract treated group and a 7.4% increase with the placebo group [29]. We reported a similar reduction in hsCRP in our earlier study with *P. emblica* in T2DM patients [14]. Similar findings were reported in the present study in which 12 week treatment with PEE250 and PEE500 showed significant reduction in hsCRP compared to baseline and placebo.

Improvement in endothelial function and dyslipidaemia achieved by amla extract seems to be brought about by a number of factors, like preventing the initiation of early ED by its antioxidant action, preventing LDL oxidation, decreasing LDL cholesterol, besides inhibiting 3-hydroxy-3-methylglutaryl- Coenzyme A (HMG Co-A) reductase activity and elevating HDL-C level [34]. The antioxidant property and cholesterol-lowering activity of polyphenolic compounds present in amla seem to play a role here.

## Conclusion

In the present study, standardized aqueous extract of *Phyllanthus emblica* significantly improved endothelial function, as evidenced by a significant decrease in reflection index, accompanied by a significant increase in NO, GSH and a significant decrease in MDA in subjects with MetS. In addition, the systemic inflammation biomarker, hsCRP, also decreased and the lipid profile improved significantly. Both 250 mg twice daily and 500 mg twice daily doses had significantly better effect than the placebo; however, the 500 mg twice daily dose was significantly more efficacious than the 250 mg twice daily dose. All the treatments were well tolerated. Hence we suggest that *Phyllanthus emblica* be used as an adjunct to conventional treatment (lifestyle modification and pharmacological intervention) in the management of MetS. Further studies in a larger population are warranted to address whether administration of *Phyllanthus emblica* may lead to a novel therapeutic alternative in improving the components of metabolic syndrome.

## Abbreviations

ADR: Adverse drug reaction
ATP III: Adult Treatment Panel III
CRP: C-reactive protein
CV: Cardiovascular
CVD: Cardiovascular disease (CVD)
DM: Diabetes mellitus
DPG: Digital volume plethysmography
DVP: Digital volume pulse
ED: Endothelial dysfunction
PEE: *Phyllanthus emblica* extract
FMD: Flow-mediated vasodilation
GSH: Glutathione
HDL –C: High-density lipoprotein cholesterol
hsCRP: High sensitivity C - reactive protein
LDL –C: Low-density lipoprotein cholesterol
LMwHTs: low molecular weight hydrolysable tannins
MDA: Malondialdehyde
MetS: Metabolic syndrome
NO: Nitric oxide
*P. emblica*: *Phyllanthus emblica*
RI: Reflection index
ROS: Reactive oxygen species
TG: Triglycerides
